# Emerging Roles of Microrobots for Enhancing the Sensitivity of Biosensors

**DOI:** 10.3390/nano13212902

**Published:** 2023-11-04

**Authors:** Xiaolong Lu, Jinhui Bao, Ying Wei, Shuting Zhang, Wenjuan Liu, Jie Wu

**Affiliations:** 1 State Key Laboratory of Mechanics and Control for Aerospace Structures, Nanjing University of Aeronautics and Astronautics, Nanjing 210016, China; bao@nuaa.edu.cn (J.B.); weiying07@nuaa.edu.cn (Y.W.); zst000@nuaa.edu.cn (S.Z.); 2 Biomedical Engineering Fusion Laboratory, The Affiliated Jiangning Hospital of Nanjing Medical University, Nanjing 211100, China; 3 College of Materials Science and Engineering, Nanjing Tech University, Nanjing 211816, China; 4 State Key Laboratory of Analytical Chemistry for Life Science, School of Chemistry and Chemical Engineering, Nanjing University, Nanjing 210023, China; wujie@nju.edu.cn

**Keywords:** microrobot, biosensor, active control, locomotion, sensitivity

## Abstract

To meet the increasing needs of point-of-care testing in clinical diagnosis and daily health monitoring, numerous cutting-edge techniques have emerged to upgrade current portable biosensors with higher sensitivity, smaller size, and better intelligence. In particular, due to the controlled locomotion characteristics in the micro/nano scale, microrobots can effectively enhance the sensitivity of biosensors by disrupting conventional passive diffusion into an active enrichment during the test. In addition, microrobots are ideal to create biosensors with functions of on-demand delivery, transportation, and multi-objective detections with the capability of actively controlled motion. In this review, five types of portable biosensors and their integration with microrobots are critically introduced. Microrobots can enhance the detection signal in fluorescence intensity and surface-enhanced Raman scattering detection via the active enrichment. The existence and quantity of detection substances also affect the motion state of microrobots for the locomotion-based detection. In addition, microrobots realize the indirect detection of the bio-molecules by functionalizing their surfaces in the electrochemical current and electrochemical impedance spectroscopy detections. We pay a special focus on the roles of microrobots with active locomotion to enhance the detection performance of portable sensors. At last, perspectives and future trends of microrobots in biosensing are also discussed.

## 1. Introduction

Biosensors featured with high selectivity and sensitivity are ubiquitous for prevailing biological analyses, showing great potential in diverse applications for detecting trace-level chemicals and biomolecules in biomedical engineering, environmental protection, and industrial fabrication, etc. [1,2,3]. For the last decades, scientists devoted to this research field have developed a number of innovative biosensors according to the diverse characteristics of tested objects [4,5,6]. Recently, it has become possible for biosensors to measure analysts in a real-time manner, which is significant for the practical monitoring of rapid changes in biological liquids [7,8,9].

In modern societies, people pay more attention to effective and affordable measurements of food safety [10], disease prevention, health status, and chronic treatment in their daily life [11]. However, for a long time, accurate detections of biological substances can only be performed in laboratories to achieve high sensitivity and low detection limits [1], which requires bulky and expensive systems that can only be operated by professional technicians [12]. Consequently, biosensors that can achieve high-quality sensing with cost-effective implementation have attracted more and more attention. Recently, the combination with personal intelligent terminals such as mobile phones and iPads makes biosensors more powerful in data processing and online diagnosis [13,14,15,16]. To meet the requirements of portable devices for easy-to-go testing, miniaturization is another crucial research focus in biosensor technology [17]. However, the miniaturization of biosensors often suppresses their detection accuracy because of the unexpectedly low efficiency of passive transport in micro volume samples. Currently, the compromise between the detection performance and miniaturization is still an unmet challenge for developing state-of-the-art biosensors.

With the fast development of nanotechnologies, the upgrade of biosensors is remarkably accelerated with rapid improvement in both sensitivity and limit of detection (LOD) [18]. Generally, biosensing nanoprobes have yielded biosensors with trace-level detection capability [17,19]. The achievements and challenges of such active machines compared with passive nanoprobes have been summarized [19,20,21,22,23]. Moreover, an attractive revolution for biosensors is on the way by using micro/nanorobots to achieve active mass transfer in the nanoscale. The micro/nanorobot which is composed of diverse functional nanomaterial can move autonomously as it is powered by chemical fuels or external physical energy sources (light, magnetic, electrical, ultrasound, etc.) [24,25]. Pioneering researches reveal the micro/nanorobot individual can work together in a collaborative manner and accomplish complex tasks including targeted delivery and nano-surgery in intricate environments [26,27]. Such minimized machines exhibit impressive motion agility and controllability, and thus they can trap the target molecules actively to achieve ultrasensitive detections [28]. In addition, the ultrasound power energy, which has been demonstrated to be harmless for biological tissues, can also be applied to actuate micro/nanorobots in a label-free and contactless way [24,29,30,31]. These driving modes determine micro/nanorobots are favorable to develop next-generation biosensors.

Focusing on the frontiers of portable biosensors combined with advanced microrobots, this review discusses their recent progress in bio-detection applications according to different assay principles, including fluorescence, surface-enhanced Raman scattering (SERS), locomotion, electrochemical current (EC), and electrochemical impedance spectroscopy (EIS). In view of the ultrasensitive detection potentials with microrobots, different manipulation methods for microrobot individuals and swarms are highlighted. Moreover, the advancement and future challenges of portable biosensors from laboratory research to practical detections are introduced to envision the prospects of the smart biosensors facilitated by micro/nanorobots. Although biosensing platforms with micro/nanorobots are still in their infancy, fantastic advantages based on active locomotion enable such smart micromachines to be an ideal partner for portable biosensors in many scenarios including accurate diagnosis, chronic disease monitoring, and explosive detections, etc., [27,32,33].

## 2. Artificial Microrobots for Biosensing

As innovative artificial machines in micro/nano scale, microrobots can be driven to operate propulsion on demand under the low Reynolds number constraints and carry payloads to move precisely under a navigation strategy for overcoming the Brownian motion [34]. With the rise and rapid development of nanotechnology, researchers have been striving to shrink the functionalized robots to cellular and molecular levels in order to perform delicate tasks in micro/nanoscale, through precise monomer or cluster control. Since then, microrobots consisting of inorganic oxides or smart materials have come into the spotlight in the field of diagnosis, sensing, microsurgery, targeted drug/cell delivery, thrombus ablation, and wound healing [35,36,37]. In 2016, the Nobel Prize in chemistry highly recognized the great potential of molecular motors, promoting the advancement and innovation of miniaturization technology. Since then, microrobots (also called micromotors, microengines, microrockets, etc.) have come into the spotlight as a powerful tool in various areas including drug delivery [38], nanosurgery, biosensing, and detoxification, which can convert diverse energies into efficient autonomous movement [39,40].

The detection methods, based on fluorescence, surface-enhanced Raman scattering (SERS), locomotion, electrochemical current (EC), and electrochemical impedance spectroscopy (EIS), have now been successfully combined with micro/nanorobots and are ready to be used in portable devices. Among these methods, fluorescence detection is widely accepted with high sensitivity and selectivity and has great advantages in detecting the presence of biomolecules. The enhanced Raman technique shows great potential in the detection of trace-level objects. The electrochemical method has the advantage of high sensitivity and fast response. In addition to the detection of biomolecules, electrical impedance also has great potential in cell detection. However, all these classic biosensing strategies are performed based on passive diffusion, which prevents their performance from being increased any more. In this context, microrobots featured with active locomotion and enrichment are demonstrated to be effective for overcoming such issues to develop next-generation biosensors with better detection sensitivities.

Beyond these five principles, it should also be noted that there are additional detection methods such as acoustic sensing and thermal sensing for biological detections. However, these types of biosensors are always used for low sensitive and harsh environments where microrobots cannot be implemented to perform accurate manipulations.

**Scheme 1:**
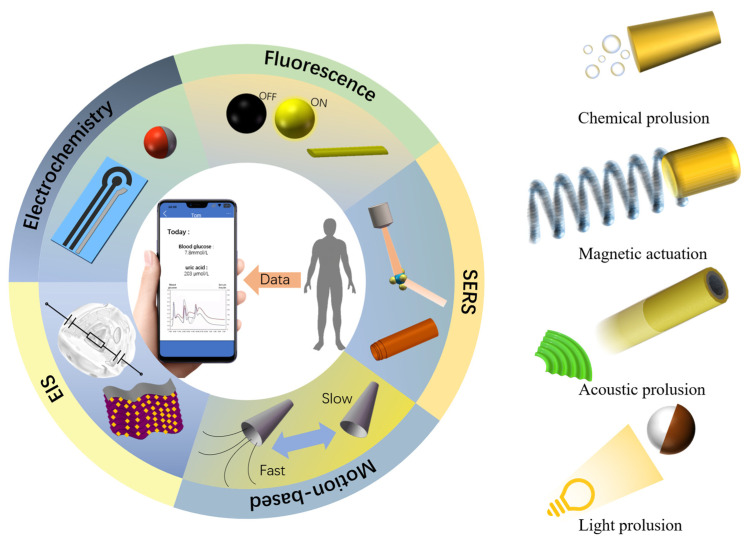
Schematic diagram for biosensors with microrobots. (SERS = surface-enhanced Raman scattering, EIS = electrochemical impedance spectroscopy).

As listed in Scheme 1 and Table 1, the applications of different types of microrobots in biosensing involve five detection principles: fluorescence detection, SERS, locomotion, EC, and EIS. The design and utilization of these microrobots offer innovative approaches to the field of biosensing. In general, the apparent geometries of microrobots are 50 nm~10 μm and their moving speed is 500 nm/s~1 mm/s. A variety of microrobots have been introduced with different materials and principles for distinct target detections. Taking fluorescence detection, for example, the fluorescence intensity needs to reach the lowest intensity for detection, which can be enhanced with the controlled motion of Janus micromotors or graphdiyne tubular catalytic microrobots [41]. The miniaturization of the fluorescence detection device has been achieved, enabling the entire detection process to be completed on site within 5 min. Microrobots made of precious metals, such as gold and silver, are also excellent probes for SERS detection to keep a close contact with analytes and on-demand enrichment, which enhanced the Raman signal approximately three times [42]. In addition, monitoring the locomotion of chemically powered microrobots is an effective mean for directly detecting chemical concentration changes [43]. Functional surface modification enables microrobots to indirectly detect substances by electrochemistry, in which the microrobot movement speeds up the electrochemical mass transfer process to improve the test sensitivity [44,45,46]. Microrobots can also detect 50 nm nanoplastics via the EIS change after their capture of target objects [47]. Most recently, acoustic microrobots have been applied into the biosensing field due to its high biocompatibility and superfast motility [48].

Since the implementation of microrobots has been conceptually demonstrated to improve the detection sensitivity of biosensors, researchers have therefore vigorously devoted themselves to developing microrobots to assist biosensing in recent years [49,50,51,52,53,54,55]. In the following part, we basically introduce the application of microrobots from five detection principles in the field of biosensing and the development of portable sensing.

**Table 1:** Summarization of typical detection principles with microrobots.

Detection Principle	Type of Microrobots ^a^	Target	Propulsion Type	Limit of Detection	Function	Refs
Fluorescence	DNA-modified Au/Pt microrobots	HIV-1 RNA	Chemical	1000 virus particles/mL	Expanding the range of target substances	[56]
	rGO/Pt microrobots	Ricin	Chemical	0.1 ng/mL	Enhance sensitivity	[57]
	rGO/Ni/PtNPs microrobots	Fumonizin B1	Magnetic	0.70 ng/mL	Reduce detection time	[58]
		Ochratoxin A		4 ng/mL		
	Graphdiyne tubular microrobots	Cholera toxin B	Chemical	1.6 ng/mL	Reduce detection time	[41]
	PEDOT/Pt microrobots	Hg^2+^	Chemical	3 mg/L	Reduce detection time	[59]
	PEDOT/SiO_2_ microrobots	Poly(lactic-co-glycolic acid)	Acoustic	-	Enhance sensitivity	[48]
SERS	Body of AgNW@SiO_2_ & tail of AgCl microrobots	Crystal violet & MCF-7	Light	-	Enhance sensitivity	[42]
	Au/SiO/Fe microrobots	Rhodamine 6G	Magnetic	0.5 nM	Enhance sensitivity	[60]
Locomotion	GO-wrapped/PtNPs Janus microrobots	Glutathione	Chemical	0.89 µM	Expanding the range of target substances	[43]
EC	Ag–AuNRs microrobots	SARS-CoV-2 virus	Magnetic	1.11 PFU/mL	Enhance sensitivity	[45]
	Ag/Fe_3_O_4_ nanorobots	SARS-CoV-2 RNA	Magnetic	6.1 ng/mL	Expanding the range of target substances	[44]
	Mg-Au Janus microrobots	Diphenyl phthalate	Chemical	0.039 mM	Expanding the range of target substances	[46]
EIS	MXene-derived γ-Fe_2_O_3_/Pt/TiO_2_ microrobots	Nanoplastics	Light	10^6^ nanoplastics/mL	Enhance sensitivity	[47]
	Mg/Fe_3_O_4_/P/anti-E microrobots	MCF-7, MCF-10A & GL261 cells	Chemical	-	Expanding the range of target substances	[61]

^a^ μ-robots = microrobots; GO = graphene oxide; rGO = reduced graphene oxide; PEDOT = 3,4-ethylenedioxythiophene; NW = nano wire; NR = nano rod; NP = nano particle.

## 3. Biosensors with Microrobots

### 3.1. Microrobots in Fluorescent Biosensing

**Figure 1:**
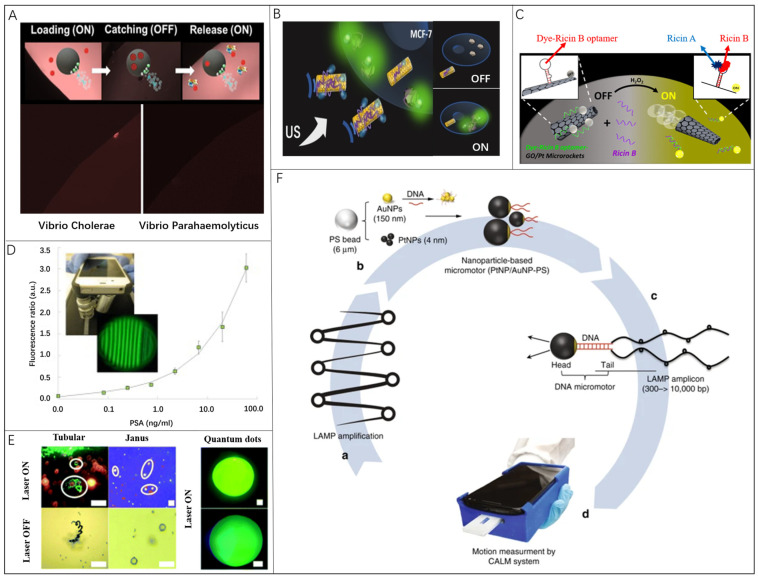
Microrobots in fluorescent bio-detection: (**A**) Janus microrobots as dynamic interfaces in the fluorescence sensing of Cholera Toxin B. Reproduced with permission [84]. Copyright 2020, American Chemical Society; (**B**) Ultrasound-propelled microrobots functionalized with fluorescein-labeled DNA aptamers for the qualitative detection of overexpressed AIB1. Reproduced with permission [70]. Copyright 2019, Wiley-VCH; (**C**) Microrobot modified with a specific ricin B aptamer tagged for detection of the ricin B toxin. Reproduced with permission [57]. Copyright 2016, American Chemical Society; (**D**) A flexible smartphone-based colorimetric and fluorescence detection system for PSA detection. Reproduced with permission [68]. Copyright 2015, Elsevier; (**E**) A smartphone-based device integrated with two kinds of microrobots for real-time fluorescence assays. Scale bars, 50 μm. Reproduced with permission [41]. Copyright 2022, the Royal Society of Chemistry; (**F**) A cellphone-based assay for HIV-1 molecular detection using loop-mediated isothermal amplification (LAMP) and microrobots. Reproduced with permission [56]. Copyright 2018, Springer Nature.

Fluorescence detection integrated with nanomaterials is a widespread approach to detect and quantify various chemicals and biomarkers [62]. The attraction of fluorescence as an analytical tool lies in the simplicity of detection, where the devices require only a closed light environment and a light detector. Variation in fluorescence intensity and color is achieved by changing the binding state of the probe to the analyte. In divergent detection scenes, various materials and detecting methods are applied in corresponding detection conditions and exhibit different levels for the limit of detection. Among these materials, graphene oxide (GO) or reduced graphene oxide (rGO) are more attractive because they can absorb the dye-labeled aptamer through π−π stacking interactions. The exceptional surface properties of graphene have allowed the attachment of different receptors for toxin detection [63]. Consequently, these receptors enable the capture of nerve agents and heavy metals [64], and then the detection can be accomplished by their fluorescence-quenching ability [65,66,67]. Additionally, one of the most widely applied fluorescence is based on the aptamer, which is taken as an excellent example of functional molecules selected in vitro [68,69,70,71,72,73,74]. More importantly, aptamers have high specificity for certain targets, ranging from small molecules to large proteins and even cells, which offers remarkable flexibility and convenience for designing biosensors with high sensitivity and selectivity [75,76,77,78]. Compared with antibodies or enzymes, aptamers have higher chemical stability and convenience in the structure design [79,80,81,82,83]. Recent researches revealed the development for designing new fluorescence (bio)-sensing strategies based on artificial microrobots. Alberto Escarpa and coworkers tested two-dimensional (2D) nanomaterials combined with microrobots in fluorescence sensing approaches [84]. As shown in Figure 1A, fluorescence-labeled probes separately wrapped by graphdiyne oxide (GDYO), graphene oxide (GO), or black phosphorous (BP) were connected to Janus microrobots with a 10 μm diameter, which can swim at 40~60 μm/s in human serum. The distinct surface functions of nanomaterials coupled with active navigation play a critical role in the loading/release capacity of the peptide, which is five times higher than the traditional method without microrobots. Such advantages greatly influence the final sensing performance for the Cholera toxin B in Vibrio cholerae and Vibrio parahaemolyticus culture samples. Subjakova et al. presented ultrasound-propelled graphene-oxide-coated gold nanowire motors functionalized with fluorescein-labeled DNA aptamers (FAM-AIB1-apt) for the qualitative detection of overexpressed AIB1 oncoproteins in MCF-7 breast cancer cells (Figure 1B) [70]. The rod-shaped gold nanorobots with an average length of ~1.7 μm and 400 nm diameter were driven by an ultrasound field to move at 25 μm/s, and the fluorescence intensity for the target protein was greatly enhanced to be four times higher. This proof-of-concept strategy for functional nanorobots has great potential for fluorescence-based sensing methods. Wang and co-workers reported an aptamer-based catalytic microrobot sensing strategy for “Off-On” real-time fluorescent detection of the ricin B toxin (Figure 1C) [57]. This approach relies on self-propelled reduced graphene-oxide (rGO)/platinum (Pt) microrobots with specific ricin B aptamer tagged with a fluorescein-amidine (FAM) dye, whose fluorescence is quenched due to π−π interactions with the rGO surface. The microrobot is ∼10 μm long and 5 μm in diameter. The influence of the microrobot’s motion state on the detection results was also studied. After incubation for three minutes, the fluorescence intensity of the static motor was 1/8 of that of the dynamic motor.

With the development of smartphones, fluorescence detection has been freed from the bondage of laboratory microscopes [10] and has become increasingly convenient for the end-users (Figure 1D) [68]. Integrated with microrobots for real-time fluorescence sensing of (bio)markers, Yuan et al. described the design of a portable device composed of a smartphone coupled with a high-resolution optical lens, custom-made emission filters, and a compartment for the insertion of low-cost commercial lasers to tailor the excitation wavelength [41]. Magnetic Janus microrobots modified with fluorescent ZnS@Cd_x_Se_1−x_ quantum dots and graphdiyne tubular catalytic microrobots modified with rhodamine-labeled affinity peptide were, respectively, used for the OFF-ON detection of mercury and cholera toxin B (Figure 1E). Draz et al. presented a platform that integrates cellphone-based optical sensing, loop-mediated isothermal DNA amplification, and microrobot motion for the molecular detection of HIV-1 (Figure 1F) [56]. The microrobots used are platinum nanoparticle (PtNP)-coated spherical polystyrene (PS) beads indirectly engineered with short DNA probes through a middle piece of spherical gold nanoparticle (AuNP). Combination with the smartphone has facilitated the miniaturization and portability of biosensors; nevertheless, there are still some shortcomings of capacities and precision compared with the results obtained in the conventional laboratory. To be specific, the acquired data show a lack of precision for quantitative detection, which calls for reliable supporting software to identify the picture and reduce the error. In addition, it is necessary to keep the detection environment as consistent as possible to reduce the impact of ambient light on the detection results. Therefore, enabling microrobots to be self-adaptable when performing long-distance enrichment based on reliable control algorithms is highly useful for the reinforcement of fluorescent signals in the next step.

### 3.2. Microrobots in Surface-Enhanced Raman Scattering Biosensing

**Figure 2:**
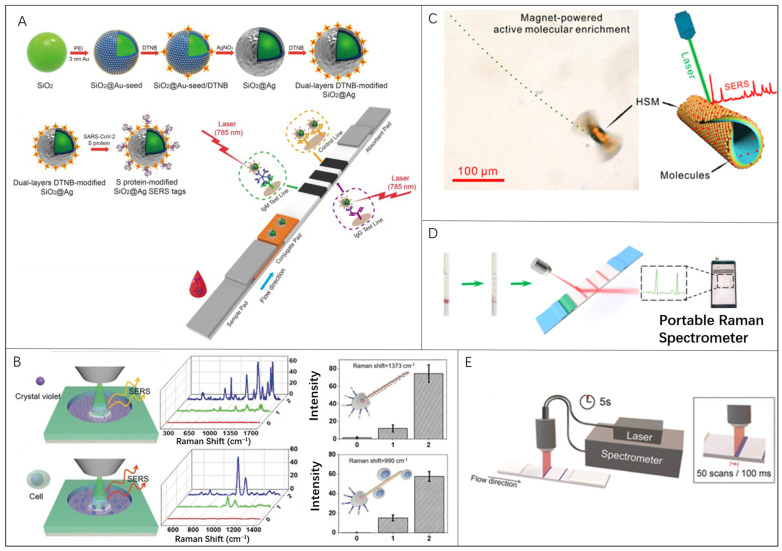
Microrobots in SERS bio-detection: (**A**) SERS-LFIA biosensor to detect anti-SARS-CoV-2 IgM/IgG. Reproduced with permission [102]. Copyright 2021, Elsevier; (**B**) Light-powered micro/nanomotor (MNM) that can serve as an active SERS probe. Reproduced with permission [42]. Copyright 2018, Wiley-VCH; (**C**) Magnet-powered tubular microrobots with high-density plasmonic nanostructure-supporting hotspots as motile hotspots for SERS sensing. Reproduced with permission [60]. Copyright 2020, American Chemical Society; (**D**) SERS-LFA strips with a smartphone-based portable Raman spectrometer for the detection of SAA and CRP. Reproduced with permission [103]. Copyright 2021, MDPI; (**E**) Portable SERS reader for rapid scanning of the test strip for detection of hCG. Reproduced with permission [104]. Copyright 2018, Wiley-VCH.

Raman scattering refers to an inelastic light scattering process that provides a vibrational spectrum representing chemical structure information [85,86,87,88]. However, the Raman scattering is a weak process, and generally the light intensity is only approximately 10^−10^ of the incident light intensity [89,90,91]. Surface-enhanced Raman scattering (SERS) is based on the enhancement effects of the rough surface of noble metals, which are 10^4^–10^7^ times stronger than traditional Raman scattering signals [92,93,94,95]. SERS is considered to be promising [96,97] as an unlabeled and rapid biosensing technology with high specificity and sensitivity through the SERS-provided vibrational spectrum information [98,99,100,101]. Liu et al. reported a surface-enhanced Raman scattering-based lateral flow immunoassay (SERS-LFIA) for the simultaneous detection of anti-SARS-CoV-2 IgM/IgG with high sensitivity [102]. Dual-layer Raman molecule-loaded Ag-coated SiO_2_ NPs (SiO_2_@Ag NPs) were used as advanced SERS tags in clinical samples to analyze anti-SARS-CoV-2 IgM/IgG (Figure 2A). The limit of detection (LOD) of SERS-LFIA for anti-SARS-CoV-2 IgM/IgG was estimated to be 1.28 × 10^7^-fold dilution by the International Union of Pure and Applied Chemistry (IUPAC) standard method, which was 800 times higher than that of standard Au nanoparticle-based LFIA for target IgM and IgG.

To improve the SERS performance, microrobots were attempted to be integrated in pioneer studies. The enhanced caption of SERS signal could be acquired by inducing adequate SERS probes in the detection area and maintaining close contact between the probes and analytes [85]. Wang et al. presented an active SERS probe of a light-powered micro/nanomotor (MNM) which has the matchlike AgNW@SiO_2_ core-shell structure (Figure 2B) [42]. The maximum speed for this type of micromotor is approximately 9 μm/s with ~30 μm length and the AgCl tail. According to micromotor enrichment remotely controlled by external light, both 10^−4^ M crystal violet and MCF-7 breast cancer cells were successfully detected with three-times-enhanced Raman signal. Using such a light-induced enrichment of the nanomotors, the Raman signals can be enhanced 6.2 times in a localized detection area in microscale as a supplement to the conventional Raman signal enhancement by SERS. Fan et al. proposed a concept of “motile hotspots” to realize ultrasensitive SERS sensing by combining hotspot engineering and active molecular enrichment. High-density plasmonic nanostructure-supporting hotspots are assembled on the tubular outer wall of microrobots via nanoimprint and rolling origami techniques (Figure 2C) [60]. The hierarchically structured microrobots (HSMs) with nanobowl- and nanocap-arrayed outer walls, respectively, show ~3.5 and ~2.2 times stronger Raman intensity when compared with the microrobot with a smooth outer wall. The size of HSMs by rolling up Au/SiO/Fe nanomembranes is affected by the thickness of nanomembranes. According to the above-mentioned methods, either by aggregating the microrobots to the target area or enriching analyte into the microrobots themselves, the improved LOD could be easily achieved.

Meanwhile, portable SERS readers for a lateral flow immunoassay (LFA) were proposed to simplify the essential complicated operations in the laboratory. Li et al. proposed a LFA strip (Figure 2D) based on SERS nanotags for the simultaneous and quantitative detection of dual infection biomarkers, serum amyloid A (SAA) and C-reactive protein (CRP), respectively [103]. Such a biosensing system achieved LODs as low as 0.1 and 0.05 ng/mL, respectively, for SAA and CRP. Tran et al. presented a Raman/SERS-LFA reader that uses a custom-made fiber optic probe for rapid, quantitative, and ultrasensitive POCT [104]. In addition, an integrated portable SERS reader had been designed and built for the rapid scanning of the test strip (Figure 2E). The pregnancy hormone human chorionic gonadotropin (hCG) is detectable in clinical samples within only 2–5 s with a LOD down to approximately 1.6 mIU/mL, which is 15 times more than a commercially available lateral flow assay. Validated by these researches, the SERS-LFA strip-based POCT technology exhibits enormous potential to satisfy the needs for simplicity and convenience in varied medical situations in the near future. Nevertheless, combining microrobots into such technology is rarely explored at this moment, which may have potential implications for POCT, telemedicine, and less-developed areas lacking in medical resources. Even though LFA is a potential development trend for portable devices, we admit that traditional laboratory clinical tests cannot be replaced and still play a critical role for accurate detection. Consequently, reliable control algorithms to realize the self-adaptable microrobot locomotion are necessary to assure the good test sensitivity for the portable SERS device.

### 3.3. Microrobots in Locomotion-Based Biosensing

The reliance of the microrobot movement on external power sources including both physical fields and chemical fuels makes it possible to design a special category of biosensors based on the relationship between the microrobot moving speed and the change in their surrounding environments [105,106,107]. Based on the working principle of microrobot motion-based detection, the concentration of the detected substance will affect the microrobot’s speed, acceleration, or deceleration [108,109,110]. Once the linear or non-linear relationship was built, the quantification detection of target reagents could be easily conducted by analyzing the motion of microrobots in real time.

**Figure 3:**
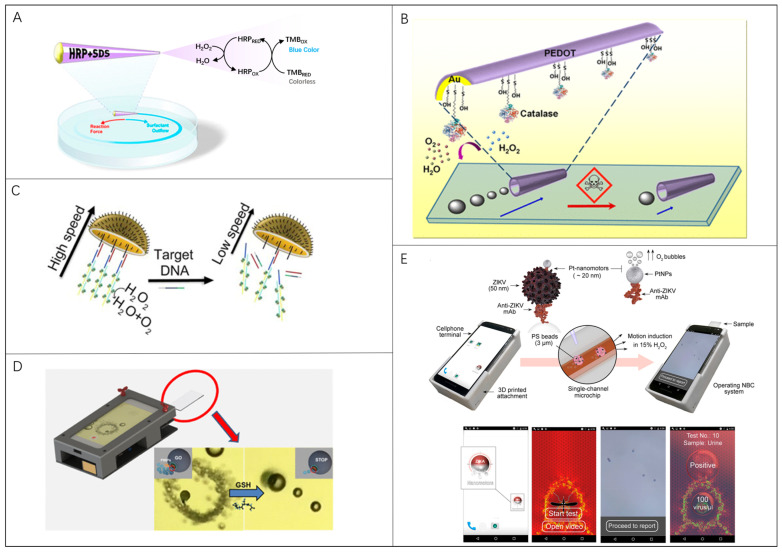
Microrobots in locomotion-based bio-detection: (**A**) Millimeter-sized tubular robots for biosensing of H_2_O_2_. Reproduced with permission [111]. Copyright 2015, American Chemical Society; (**B**) Enzyme-powered polymeric (PEDOT)/Au-catalase microrobots for water-quality testing. Reproduced with permission [112]. Copyright 2013, American Chemical Society; (**C**) Chemically powered jellyfish-like microrobots for detection of DNA which slow down the microrobot. Reproduced with permission [113]. Copyright 2021, American Chemical Society; (**D**) A smartphone-based platform for motion-based detection of GSH which reduces the speed of microrobots. Reproduced with permission [43]. Copyright 2018, American Chemical Society; (**E**) Immunological detection of ZIKV using Pt-nanorobots and bead motion monitor of a cellphone optical system. Reproduced with permission [114]. Copyright 2018, American Chemical Society.

Moreno-Guzman et al. reported a one-millimeter-sized tubular micromotor for mobile biosensing of H_2_O_2_ in environmental and relevant clinical samples (Figure 3A) [111]. Sodium dodecyl sulfate (SDS) surfactant and horseradish peroxidase were released from the rear of the microrobot which was propelled by the Marangoni effect. In this case, the motion of a single millimeter-sized tubular micromotor for 120 s was measured to quantify the concentration of H_2_O_2_ in different samples. Similarly, Orozco et al. presented a novel microrobot-based strategy for water-quality testing based on changes in the propulsion behavior of artificial biocatalytic microswimmers influenced by aquatic pollutants (Figure 3B) [112]. The presence of 100 μM Hg leads to a rapidly diminished propulsion efficiency with speed diminutions of 90–95% for enzyme-decorated PEDOT/Au millimeter-sized tubular micromotors within 6 min. Zhang et al. fabricated a chemically powered jellyfish-like microrobot by using a multimetallic shell, of which a DNA assembly with catalase decorations was modified on the concave surface to simulate the umbrella-shaped body and the muscle fibers on the inner umbrella of jellyfish (Figure 3C) [113]. The microrobot is a hemispherical shell structure with an opening of 20 μm and can swim with speed, exceeding 209 μm/s in 1.5% H_2_O_2_. Once the target DNA hybridizes with the microrobot’s DNA, H_2_O_2_ catalase falls off and slows the microrobot down.

Moreover, the optical equipment is essential for observing microrobot movement. Portable devices with a combination of a smartphone and an optical lens show great superiority compared with the traditional lab-used bulky microscopes. Yuan et al. built a Janus microrobot platform for the motion-based detection of glutathione by coupling an ordinary smartphone with an external magnification optical lens. (Figure 3D) [43]. The speed of 20 μm graphene-wrapped/PtNPs Janus microrobots was correlated with the concentration of glutathione, which was utilized to detect the presence of glutathione with a limit of detection (LOD) of 0.90 μM. Draz et al. reported a nanomotor-based bead-motion cellphone (NBC) system for the immunological detection of the Zika virus (ZIKV) [114]. Pt-nanomotors with a ~20 nm diameter driven by catalyzing H_2_O_2_ could be attached to the beads under the action of ZIKV (Figure 3E). Thus, the average motion velocity of the beads was quantitatively correlated to the virus concentration in the tested sample. The employed smartphone with an optical accessory is also equipped with a customized program to track the bead’s movement, measure the moving velocity, and calculate the virus concentration. Obviously, mass production for high-performance microrobots with good locomotion capabilities should be given the first priority to explore for developing new synthesis strategies.

### 3.4. Microrobots in Electrochemical Current-Based Biosensing

**Figure 4:**
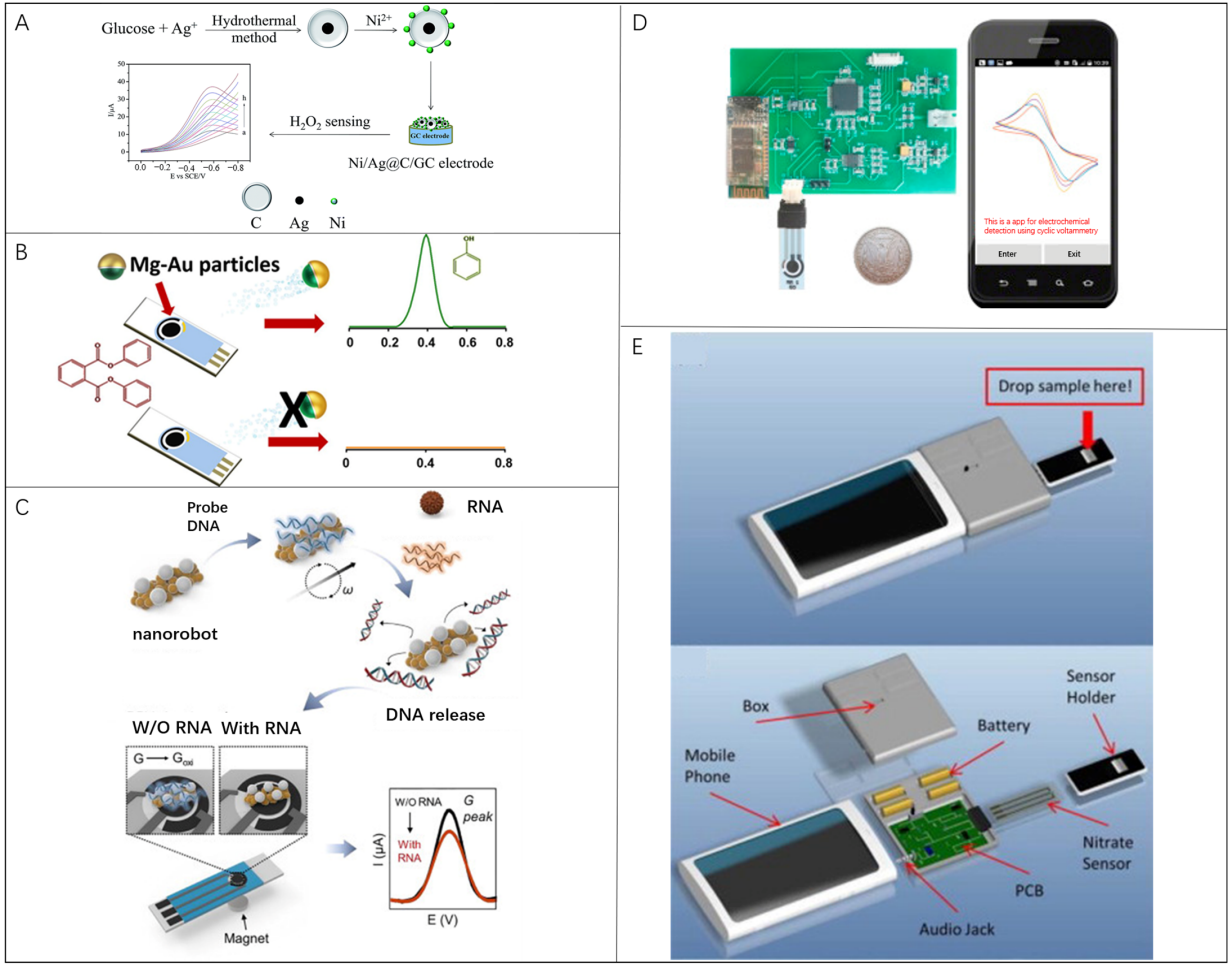
Microrobots in electrochemical-current bio-detection: (**A**) H_2_O_2_ sensor modified with the Ni-doped Ag@C (Ni/Ag@C) nanocomposites on the surface of a glassy carbon electrode. Reproduced with permission [126]. Copyright 2017, the Royal Society of Chemistry; (**B**) DPP detection strategy based on Mg/Au Janus micro-robots. Reproduced with permission [46]. Copyright 2016, American Chemical Society; (**C**) Plasmonic-magnetic nanorobot-based COVID-19 detection assay. Reproduced with permission [44]. Copyright 2022, Elsevier; (**D**) Smartphone-based CV system consisting of modified electrodes, a portable electrochemical detector, and a smartphone. Reproduced with permission [123]. Copyright 2017, Elsevier;. (**E**) Mobile phone sensing platform to detect nitrate concentration. Reproduced with permission [128]. Copyright 2015, Elsevier.

Apart from optical methods that rely on the microscope- or smartphone-integrated magnify lenses, electro-signal-based detection offers another option to build portable biosensors independent of the delicate optical system, and thus is highly suitable for incorporating with existing MEMS devices [115,116,117,118]. The electrochemical detection reads the current signal generated by the redox reaction of the analyte on the electrode to obtain the concentration of the analyte [51,119]. As one of the most important methods in electroanalytical chemistry, cyclic voltammetry (CV) is often applied in electrochemical current detections [46,120,121,122,123,124,125,126,127,128,129,130,131]. For example, the electrochemical sensing strategy reported by Sheng et al. for H_2_O_2_ concentration measurement followed this basic principle [126], of which the working electrode was modified with Ni-doped Ag@C (Ni/Ag@C) nanocomposites (Figure 4A). The performance of the sensor was studied by cyclic voltammetry measurement of H_2_O_2_ to illustrate its LOD down to 0.01 mM.

The addition of microrobots in electrochemical detection accelerates the ambient liquid flow, so as to improve the detection sensitivity and LOD. Rojas et al. presented a novel Janus microrobot-based strategy for the direct determination of diphenyl phthalate (DPP) in food and biological samples. Mg/Au Janus microrobots (average diameter, 20 μm) degraded DPP to phenol, which is directly measured by difference pulse voltammetry on disposable screen-printed electrodes (Figure 4B) [46]. Simultaneously, in the absence of microrobots, the electrochemical reaction signal has not been observed. Efficient swimming of multiple Mg/Au micromotors in complex samples introduce a 20-fold increase in the sensitivity of DPP detection compared with that in static conditions. The average speeds of microrobots are 108 ± 18, 296 ± 40, 223 ± 38, and 40 ± 8 μm/s in milk, water, whiskey, and serum samples, respectively. Kim et al. showed another plasmonic–magnetic nanorobot-based simple but efficient COVID-19 detection assay through an electronic readout signal (Figure 4C) [44]. With a movement controlled by the magnetic field, nanorobots composed of Fe backbone and Ag surface were used to transport and release the probe nucleic acid. The microrobots with a length of 2.49 ± 0.59 μm, and width of 1.10 ± 0.28 μm, can move at a maximum speed of 8.9 μm/s. This strategy has universal adaptability and can be extended to various nucleic acid detections.

Similar to the optical counterparts, electrochemical detection based on smartphones has also been reported. Smartphones serving as data processors and displayers in electrochemical detection have a great potential to combine advanced 5G technologies. Ji et al. designed a smartphone-based CV system for portable detection as shown in Figure 4D. The system consisted of screen-printing modified electrodes, a portable electrochemical detector, and a smartphone [123]. The reduced graphene oxide (rGO) and 3-amino phenylboronic acid (APBA) were modified on the screen-printed electrodes for detection. The LOD for glucose was approximately 0.026 mM with test errors less than 3.8% compared with the commercial electrochemical workstation. Wang et al. proposed a mobile phone sensing platform for nitrate in water by electrochemical measurements using the audio jack function (Figure 4E) [128]. The platform utilized a cyclic-voltammetry-based electrochemical process with a LOD of 0.2 ppm within 1 min. In this work, the influence of external interference factors on detection stability was eliminated, such as temperature, pH, and ion interference conditions. Compared with commercial electrochemical workstations, portable electrochemical sensors hold an acceptable measuring error with a smaller equipment volume and lower cost. Considering the possibility exhibited by micro/nanorobots in electrochemical detections, diversified modification of individual micro/nanorobot and further exploration to utilize the motility of micro/nanorobots could pave a new way to improve the portability and detection performance of next-generation portable electrochemical biosensors. Under this situation, we envision that it is valuable to clarify an accurate swarm cooperation mechanism to overcome disturbance from the harsh chemical environment.

### 3.5. Microrobots in Electrochemical Impedance Spectroscopy Biosensing

By means of measuring the intrinsic electrical properties of the target in the electric field, electrochemical impedance spectroscopy (EIS) offers a biocompatible and harmless methodology for detecting various items such as bacteria, biological cells, and tissues, etc., [132,133,134,135]. Though EIS has an unmatched advantage in cell identification by measuring the impedance signal corresponding to distinct cell lines in a biocompatible way, excluding toxic reagents, the precision of detection is highly dependent on the location of the cell during the detecting procedure or the data calibration process. Introducing microrobots and their motion control systems can convert the existing unmanageable cell passage into a controllable route and significantly improve the detection performance.

**Figure 5:**
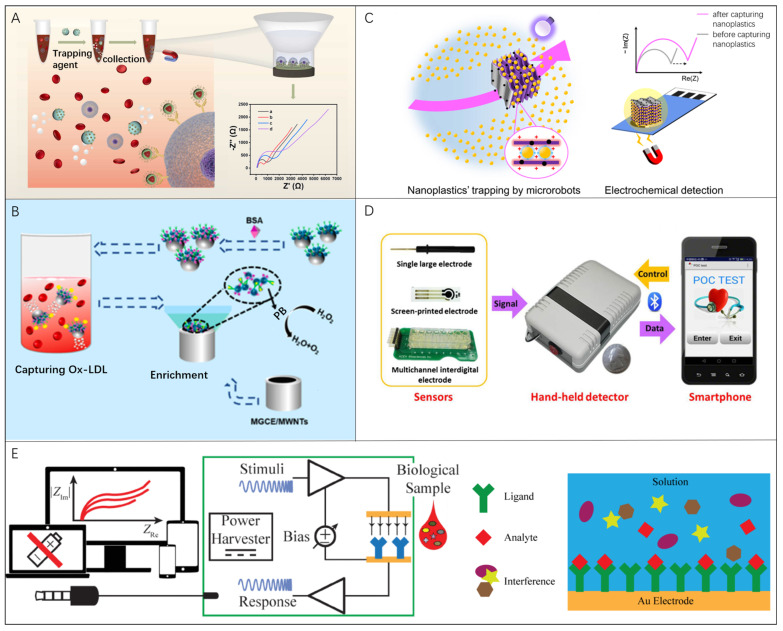
Microrobots in EIS bio-detection: (**A**) A funnel-shaped device to realize the EIS detection of captured CTCs based on Mg-based microrobots. Reproduced with permission [61]. Copyright 2023, Elsevier; (**B**) An EIS sensor platform based on microrobots to actively capture and detect Ox-LDL in whole blood. Reproduced with permission [136]. Copyright 2017, Elsevier; (**C**) Nanoplastics’ detection by EIS using self-propelled microrobots for preconcentration in an electrolyte solution (yellow shading). Reproduced with permission [47]. Copyright 2014, Springer Nature; (**D**) Smartphone-controlled biosensor system to detect proteins for POCT, which was constituted by the miniaturized biosensor, hand-held EIS detector, and smartphone. Reproduced with permission [137]. Copyright 2016, Elsevier; (**E**) Mobile health immunoassay platform based on audio-jack-embedded devices in which the sensing electrode is modified with a ligand complimentary to bind analyte. Reproduced with permission [138]. Copyright 2022, Elsevier.

Wan et.al realized an efficient detection of circulating tumor cells (CTCs) through EIS means utilizing Mg-based microrobots (Figure 5A) [61]. Using an aldehyde–amine condensation reaction, the Mg-based microrobots can be modified with Fe_3_O_4_/P/anti-E nanoparticles to capture CTCs. With the hydrogen (H_2_) propulsion by Mg reaction, microrobots maintain 16.5 μm/s motion to increase the chance of anti-E recognition and capture of CTCs. The EIS detection platform based on Mg-based microrobots have a good linear response range and low detection limit for CTCs in untreated blood samples (~5 cells/mL). This method was also demonstrated to be effective for detecting oxidized low-density lipoprotein (Ox-LDL) in whole blood just by replacing anti-E components with the antibody of Ox-LDL for microrobots (Figure 5B) [136]. Due to the application of microrobots, the electrochemical sensor shows a good detection for Ox-LDL with range from 1 × 10^−2^ μg/mL to 10 μg/mL and the LOD of 9.8 × 10^−4^ μg/mL. Most recently, the inducement of microrobots offers another choice to aggregate the nanoplastic pollutions in an active manner. Urso et al. developed the multifunctional MXene-derived oxide microrobots that helped to collect randomly distributed nanoplastics in the three-dimensional (3D) space onto the sensing electrodes for EIS detection (Figure 5C) [47]. Compared with the modification of electrodes, well-designed microrobots show not only the capability to trap the target within the whole volume of the liquid sample but also the controllability to locally concentrate the target effectively.

Portable electrical impedance analyzers rely heavily on smartphones for data analysis and display. Zhang et al. developed a smartphone-controlled biosensor system that consisted of a miniaturized biosensor, a hand-held EIS detector, and a smartphone to quantify different kinds of proteins by exchanging electrodes for POCT [137]. The smartphone provides control commands and receives data signals via Bluetooth, meanwhile acting as a displayer for the measurement results in form of a Nyquist plot (Figure 5D). The detection limits of bull serum albumin (BSA, also known as bovine serum albumin) and thrombin are 1.78 μg/mL and 2.97 ng/mL, respectively. Jiang et al. presented a mobile health immunoassay platform based on audio-jack-embedded devices, such as smartphones and laptops, which uses EIS to detect a NeutrAvidin self-assembled monolayer [138]. Self-assembled monolayers have been immobilized on gold electrodes. A complementary ligand of the target analyte is modified on the sensor electrode to cause impedance changes when combined with the detecting target (Figure 5E). Regarding the fast development of a wearable and portable biosensing device, it is essential to carry out auxiliary instrument minimization for driving microrobots for practical implementation.

## 4. Conclusions and Perspective

In this review, we discussed the combination strategies of innovative microrobots with biosensors according to five classic principles, including fluorescence detection, SERS detection, microrobot motion-based detection, electrochemical-current detection, and EIS detection. Well-designed micro/nanorobots assist conventional biosensing systems to adapt to various complex scenarios at higher sensitivity and lower LOD. In addition, the emergence of microrobots is crucial for indirectly detecting trace-level biological matters hardly detected before. Researchers often design and synthesize microrobots for specific applications based on their research goals. Considering the existing circumstances, it is still a long way to go for commercially available microrobots for users. Despite the fact that the presented merits are fantastic for actively enhancing biosensor performances, there is still plenty of room at the bottom for microrobots to be further explored from the following aspects:

(i) Development of new synthesis strategy and surface modification to rise the loading capacity. Microrobots serving as active probes for biosensing tests are mostly fabricated with expensive materials and the surface modification is time consuming. Creating microrobots that use inorganic materials should be more efficient for mass production. Solvothermal/hydrothermal methods and template-assisted electrodeposition are adopted to synthesize microrobots just by one-step fabrication. Moreover, a large number of inorganic microrobots are featured with porous structures, which are better suited for rising the loading capacity. Based on the capability of mass production and enough space for surface modification, inorganic microrobots become excellent candidates to be applied in biosensor systems.
(ii) Accurate swarm cooperation mechanism to overcome disturbance from harsh environments. Due to the high viscosity and large concentration of ions of tested specimens, microrobot individuals are difficult to effectively move because of the weak propulsion force. Based on swarm cooperation, one group of microrobots can aggregate together to output a large propulsion force together to overcome the disturbance. In general, the microrobot swarm powered by ultrasound maintains the highest locomotion speed and thus are primarily considered to be implemented for harsh environments in the biosensor platform. However, the method for accurately controlling the superfast movement of the ultrasonic microrobot swarm is lacking. With the development of metamaterials, unusual physical fields can be generated to modulate the spatial intensity and temporal variation, which can further improve the control accuracy of different microrobot swarms.
(iii) Auxiliary instrument minimization for driving microrobots. It becomes easy for ordinary people to obtain commercialized biosensors, which are always designed to be portable or wearable for daily use. However, the operation of microrobots requires many auxiliary instruments closely related to the working principles. Most instruments for physically powered microrobots are bulky and expensive, and can only be implemented in the laboratory. With advanced electronic integrated circuit technology, it is possible to shrink these components to a minimized size, which can be well matched with the portable biosensing system. Regarding the costs for commercialization, detachable driving instruments are feasible if the microrobots can be effectively maneuvered to work inside biosensors.
(iv) Reliable control algorithms to realize the self-adaptable microrobot locomotion. Currently, microrobots can be controlled to perform 2D in-plane movement, but long-distance locomotion along intricate routes is still not yet well demonstrated. For biological applications, multi-functional tests for different properties are sequentially accomplished on the same piece of biosensor with distributed testing regions. The fixed program setting is impossible to control microrobots to move effectively from one region to another in mazy microchannels. An artificial neural network control algorithm is a good option to be integrated with the manipulation system for dynamically optimizing the driving signals, enabling microrobots to be self-adaptable when performing multiple tasks in long-distance locomotion.

Even though many practical troubles of applying microrobots to enhance biosensors is still unsolved and a number of challenging issues should be addressed, we believe that biocompatible, eco-friendly, and dexterous microrobots will become reliable and powerful partners for versatile biosensors to better serve diverse application scenarios in the future.

## Data Availability

The authors confirm that the data supporting the findings of this study are available within the article.
